# Supplementary material for: Effect of land-use practices on species diversity and selected soil property in Somodo Watershed South-Western Ethiopia
Source: PLoS One. 2021 May 28;16(5):e0252305. doi: 10.1371/journal.pone.0252305 (PMC8162689; doi:10.1371/journal.pone.0252305)
Supplement: Attachment — Submitted filename: Reviewers comment.docx [file pone.0252305.s002.docx]

| **Section** | **Reviewers’ comment** | **Author’s response** |
| --- | --- | --- |
| General comment: | (i) Provide the site characteristics including slope, aspect, vegetation characteristics for each of the land-uses selected for the study. | The issue taken into account and necessary correction was under taken. |
|  | (ii) Provide the original reference for the IVI | Original reference is not clear for us. However, the data collected from the field is used for comparison of species diversity under different land use |
|  | (iii) Correct the spelling of Jackson 1958 | Corrected - |
|  | (iv) Provide the axis title for the Figure 3. | Corrected accordingly |
|  | (v) Add a new section on the implication of the study | Considered and the implication of the study presented in short summary |
