# Supplementary material for: Effect of land-use practices on species diversity and selected soil property in Somodo Watershed South-Western Ethiopia
Source: PLoS One. 2021 May 28;16(5):e0252305. doi: 10.1371/journal.pone.0252305 (PMC8162689; doi:10.1371/journal.pone.0252305)
Supplement: Attachment — Submitted filename: Response to Reviewers.docx [file pone.0252305.s003.docx]

| **Date:** | March 14, 2021 |
| --- | --- |
| **To:** | " PLOS ONE" plosone@plos.org |
| **From:** | " Leta Hailu Gemechu" latahailu@gmail.com |
| **Subject:** | Authors: Response to reviewer [PONE-D-20-33362R1] |

**Dear Editor-in Chief**

We are very grateful to the reviewer for his valuable and constructive comments to our manuscript entitled “Effect of Land-use Practices on Species Diversity and Selected Soil Property in Somodo Watershed South-Western Ethiopia, ms number “PONE-D-20-33362R1”. We have attempted to address all comments in this revised manuscript and now the manuscript is greatly improved. We highlighted **yellow colour** on the **“revised Manuscript with Track Change”** for highlights additional inputs and changes made to the original version. The responses given for comments are highlighted **red colour** in Authors’ responses page. Below are our responses to the specific comments raised by the reviewer # 1.

1. Reviewer #1: The manuscript titled “Effect of Land-use Practices on Species Diversity and Selected Soil Property in Somodo Watershed South-Western Ethiopia” was reviewed and found that the authors have not written it with clarity. Grammatical and typographical errors are numerous to list. Scientific names of plant species need to written in italics. The manuscript is not suitable for publication in its current form. Authors are advised to revised it critically and resubmit. The following observations may also be addressed adequately while revising the manuscript. Thank you for your valuable comments, suggestions and in depth review of the manuscript. We endeavor to address all the given comments and suggestions accordingly to improve the manuscript.
1. P1 L10: “the” land-use practices, thank you for your suggestion, now corrected with appropriate punctuation (the land-use practice)
2. P1 L12: ‘trail walk’ or ‘transect line’ instead of ‘transect walk’, (10 ×10) m2. Thank you, now corrected to transect line & (10 ×10) m^2^
3. P1 L13, 14 & 15: “were sampled from plots exhibiting different land-use practices found in the watershed in order to evaluate species richness and diversity. Soil samples were also collected from each plot. The soil samples were analyzed following standard laboratory procedures. Thank you for your suggestion and the comments considered.
4. P1 L15: shows showed. Corrected.
5. P1 L16: between among. Now corrected.
6. P1 L17 & 18: Coffea arabica was dominant in homestead gardens and natural forests while Giravilia robusta showed had maximum richness in plantations and farm forests in Somodo Waatershed. Now corrected accordingly.
7. P1 L19-21: There is a lack of clarity in interpretation and presentation of major findings of the study. Abbreviations need to be explained and results must appear before moving on to deductions. Please write full form of OC, TN and C: N in the abstract or in its first appearance. The abstract is completely descriptive, it requires to be written highlighting the key findings on species diversity, richness and soil parameters analysed in the study. Thank you for your constructive comments and now improved accordingly.
8. Comment on L 10-25: The abstract requires a grammatical check. Proper data-oriented results should lead to deductions and conclusions. Thank you for your comment, now tried to improve
9. P2 L 26: several degrees various extents. Now replaced with the suggested one
10. P2 L37:
    • exacerbates aggravates
    • This implies that the reversal of land degradation through better understanding of local proximate and underlying drivers. Now updated with the suggested one
11. P2 L40: to different degrees. Now corrected
12. P2 L36-44: Implications of words like “that”, “these” are not clear. It shall be better if it is replaced by scientific terminologies or methodologies as the case may be. Thank you for the comment, now replaced with the correct one
13. P3 L 61: “in” the upper part of Dhidhesa catchment. Considered.
14. P3 L 72: account accounting. Now updated with suggested one.
15. P4 L 73 & 74:and the pH of the soil is rated as strong acidity. while the pH is found to be strongly acidic. Now corrected with the suggested one.
16. Figure 2: The content of the ‘description of study area’ do not reveal clear correlation between rainfall and temperature. Therefore, it is advised to use two separate frequency line graphs depicting patterns of annual rainfall and temperature (max & min). Thank you for the suggestion, here, the objective is to show the trend and long term mean of rain fall, min & max temperature of the area.
17. P4 L 77 & 78: What is meant by “and agro-forestry practices”? It needs to be clarified in context to the prevailing land use practice. Now corrected to home garden agroforestry which is stands for the prevailing land-use in the study.
18. P4 L 82: Capitalize ‘several’ to ‘Several’. Now corrected to the suggested one.
19. P4 L82-87: The paragraph requires careful grammatical check. The words ‘dominated’ and ‘conquered’ have been interchangeably used, which makes it unclear. Use of proper punctuations at the correct places is advised, the lack of which renders the sentence a different meaning. Sure, the comments are considered and corrected accordingly.
20. P5 L 90: Maize maize. Now corrected
21. P5 L94 & 95: their main source of income is gained from the selling of their main source of income is obtained from the sale of crops, fruits and livestock. The use of coordinating conjunctions is not clear in this paragraph. Thank you for the comment, now corrected according to the suggested one.
22. P5 L 96-109:
    • Field observations were held along a transect line with plots measuring (10 × 10) m2. was established along the transect line as the land-uses appear. Now corrected.
    • Namely should be replaced by ‘viz.’ Now corrected.
    • Soil sampling technique using augar should follow some protocol. Thank you for the comment soil sample collection using auger is possible up to 0 to 30 cm.
23. P5 L 111: Since the term, ‘soil reaction’ has not been used before this; it is advisable to write it as ‘pH of soil’. Now corrected.
24. P6 L 114: This line mentions processes which require citation. Thank you for the comment, I think the citation is already there.
25. P5 L119: The use of “the” needs to be checked. Considered
26. P5 L 121: ‘Microsoft’ Excel ‘version?’. Thank you, now corrected.
27. P5 L122: General linier model generalized linier model. Thank you, corrected.
28. P5 L 124: What is meant by “for windows”?
29. P5 L124: LSD is Least significant difference not list significant difference. Now Corrected.
30. P5 L125: when they are significantly different (p≤0.05). corrected.
31. P 5 L 128: characterized categorized.
32. P 5 & 6 L 127-137:
    • Scientific names need to be written in italics following taxonomic norms.
    • Use of proper punctuations is required. Thank you for the comment, the suggestion is considered.
33. P 6 L 138-145:
    • Proper framing of sentences coupled with the use of proper punctuations and a grammatical check is suggested. Sure, considered.
    • Also, look into the plagiarism of this paragraph.
    • Singular and plural forms should be properly deployed. Sure, considered.
    Figure 3 should be better represented with a pie chart. Thank you for your suggestion.
34. P 8 L 149-157:
    • Grammatical check for this paragraph is suggested.
    • Comparisons from figure 3 are not clear. Thus, proper use of wordings and punctuations are suggested. Thank for the suggestions, now corrected.
35. P 8 L 158 – 164:
    • Please mention the significance level of testing in line number 158-159. Thank you for the comment, now mentioned.
    • Use of proper punctuations is desirable in case of comparison statements. Thank you, now corrected.
36. P 8 L 165: Kindly mention the significance level for hypothesis testing.
37. P 9 L 173 & 174: Total Nitrogen (T.N.) did not exhibit significance difference (at level of significance?) among various land-use practices in the study watershed. Now the significance level is mentioned and the suggestion is accepted accordingly.
    Table 1: Please maintain uniformity in alignment of data in table. Now the uniformity of the data in the table is corrected.
38. P 9 L 190: coffee Arabica Coffea arabica. Now corrected.
39. P 9 L 191: Concerning this In contrast/ Compared to this. Now updated with the suggested one.
40. P 10 L 197 – 198: Please write scientific names in italics following taxonomic norms. Now corrected.
41. P 10 L 204: The study suggests This study suggest. Corrected accordingly.

Many thanks for your constructive and valuable comments!!
