# Supplementary material for: Effect of land-use practices on species diversity and selected soil property in Somodo Watershed South-Western Ethiopia
Source: PLoS One. 2021 May 28;16(5):e0252305. doi: 10.1371/journal.pone.0252305 (PMC8162689; doi:10.1371/journal.pone.0252305)
Supplement: Attachment — Submitted filename: Response to Reviewers.docx [file pone.0252305.s004.docx]

| **Date:** | April 23, 2021 |
| --- | --- |
| **To:** | " PLOS ONE" plosone@plos.org |
| **From:** | " Leta Hailu Gemechu" latahailu@gmail.com |
| **Subject:** | Authors: Response to reviewer [ PONE-D-20-33362R2] |

**Dear Editor-in Chief**

We are very grateful to the reviewer for his constructive and positive comments to our manuscript entitled “Effect of Land-use Practices on Species Diversity and Selected Soil Property in Somodo Watershed South-Western Ethiopia, manuscript number “PONE-D-20-33362R2”. We have tried to address all comments in this revised according to reviewer and academic editor comments and suggestion. We hope now the manuscript is fit the journal publication standards. We highlighted **yellow and red colors** on the **“revised Manuscript with Track Change and Figure 3”** for highlights additional inputs and changes made to the original version. The responses given for comments are highlighted **red colour** in Authors’ responses page. Below are our responses to the specific comments raised by the reviewer # 1.

Reviewer #1: The revised version of manuscript ID PONE-D-20-33362R2 was reviewed and found that authors have improved it up to a significant extent possible. Authors are advised to correct scientific name of plant species in Figure 3 as well. All names must be written in italic.

Thank for your comments and suggestions, we considered the comments accordingly and all scientific names checked for its correctness and made in italic style.

1. Gravillia Robusta- Grevillea rubusta
2. Eucalyptus- Eucalyptus camaldulensis
3. Erthirina abyssinica- Erthrina abyssinica
4. Vernonia schimperi- Vernonia amygdalina
5. Kalanchee petitana- Kalanchoe petitiana
6. Sysygiumguineense – Sysygium guineense

Editor Note: please upload your figure files to the Preflight Analysis and Conversion Engine (PACE) digital diagnostic tool

All figures are uploaded to PACE and corrected accordingly and attached with zipped file.

Many thanks for your constructive and valuable comments!!
